# Interaction of MMP-9 in the active phase of Graves’ disease with and without ophthalmopathy

**DOI:** 10.1152/ajpendo.00166.2024

**Published:** 2024-09-11

**Authors:** Cinthia Minatel Riguetto, Eduardo Buzolin Barbosa, Camila Cristina Atihe, Fabiano Reis, Monica Alves, Denise Engelbrecht Zantut-Wittmann

**Affiliations:** ^1^Endocrinology Division, Department of Internal Medicine, Faculty of Medical Sciences, University of Campinas, São Paulo, Brazil; ^2^Department of Ophthalmology and Otorhinolaryngology, Faculty of Medical Sciences, University of Campinas, São Paulo, Brazil; ^3^Department of Radiology, Faculty of Medical Sciences, University of Campinas, São Paulo, Brazil

**Keywords:** Graves’ disease, Graves’ ophthalmopathy, IL-17a, MMP-9, thyroid eye disease

## Abstract

Thyroid eye disease (TED) is expressed as orbital inflammation, and serum levels of several proinflammatory cytokines have been studied among patients with Graves’ disease (GD) with and without TED; however, a more sensitive and specific marker for the different phases of GD and TED is still lacking. Seventeen active TED, 16 inactive TED, 16 GD without TED, and 16 healthy controls were recruited. Serum IL-17A, MMP-2, MMP-3, and MMP-9 were measured by multiplex bead assay. TED hormone and eye parameters were evaluated, and their relationship with cytokine levels was analyzed. Serum MMP-9 was higher in active TED than healthy controls, whereas IL-17A was lower among these patients than in GD without TED and healthy controls. No differences were found in MMP-3 and MMP-2 concentrations. MMP-9 levels were lower in patients with inactive TED who underwent radioactive iodine (RAI) therapy and those on levothyroxine replacement. MMP-9 levels were elevated in patients on methimazole. A negative correlation was found between age at assessment and time of follow-up with MMP-9 levels in inactive TED. Free T3 and ophthalmometry values were positively correlated with MMP-9 in the GD without TED and inactive TED groups, respectively. In conclusion, serum MMP-9 was increased in patients with active TED and was related to the RAI treatment, longer follow-up time, and higher ophthalmometry in patients with inactive TED, as well as thyroid function in GD without TED. MMP-9 may be involved in both the active phase of TED and the active phase of inflammation related to GD.

**NEW & NOTEWORTHY** Our study addresses clinical aspects of specific ophthalmological examination and serum cytokine concentrations of patients with Graves’ disease (GD) with and without ophthalmopathy. Our findings suggest that MMP-9 may be involved in the active phase of ophthalmopathy and in the active phase of GD. The central question is whether MMP-9 is a potential target for future treatments.

## Graphical abstract

**Figure:**
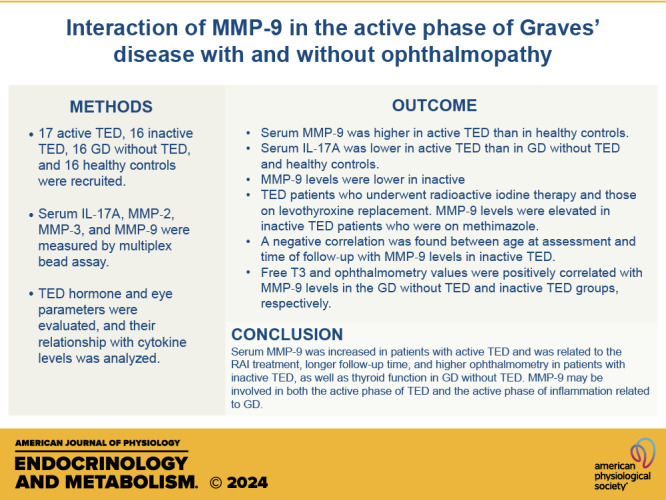

## INTRODUCTION

Graves’ ophthalmopathy (GO) is the most common extrathyroidal manifestation of Graves’ disease (GD), expressed as orbital inflammation and expansion of extraocular fat and muscles (1). This condition is also called thyroid eye disease (TED) and consists of an initial acute inflammatory phase followed by a chronic fibrotic course, characterized by type 1 helper T cells (Th1) and type 2 helper T cells (Th2) cell immune responses, respectively. The Th1 response is marked by cell-mediated immunity and the production of large amounts of interferon-gamma (IFN-γ), tumor necrosis factor-alpha (TNF-α), interleukin 1 beta (IL-1β), and interleukin 2 (IL-2), whereas Th2 cells primarily activate humoral reactions, producing interleukin 4, 5, 10, and 13 (IL-4, IL-5, IL-10, and IL-13) (2, 3).

In addition to Th1 and Th2, a subgroup of T cells named Th17 cells produces IL-17, which, given its proinflammatory profile, has been associated with several autoimmune disorders (4). Most studies have reported a positive correlation between IL-17 concentrations in patients with TED and the clinical activity score (CAS) (5, 6).

Matrix metalloproteinases (MMPs) are a group of zinc-dependent endopeptidases involved in the degradation and extracellular matrix (ECM) remodeling under physiological and pathological conditions that have also been recently studied in GD with and without TED (7, 8). Among more than 20 different types of MMPs, MMP-2, MMP-3, and MMP-9 seem to promote leukocyte migration in animal models, possibly acting in acute and chronic phases of inflammation (8, 9). Kapelko-Stowik et al. (10) reported higher levels of MMP-2 and MMP-9 among patients with GD with and without TED than healthy subjects. MMP-9 was also positively correlated with CAS. On the other hand, Mysliwiec et al. (11) demonstrated that only serum MMP-9, not MMP-2, significantly decreased after glucocorticosteroid therapy in GO.

Although several proinflammatory cytokines have been more extensively studied among patients with GD with and without TED, a more sensitive and specific marker for the different phases of GD and TED is still lacking. Hence, in this study, we aimed to analyze the profile of serum cytokines such as MMP-2, MMP-3, MMP-9, and IL-17A among patients with GD without apparent TED and those with TED in active and fibrotic phases of inflammation.

## MATERIALS AND METHODS

### Study Subjects

This was a cross-sectional clinical study, part of a larger study conducted at the University of Campinas (UNICAMP) between August 2019 and March 2020. All participants provided written informed consent, and ethical committee approval for the study was obtained according to the Declaration of Helsinki, Human Research Ethics Committee in Lausanne, No. 204/14). Written informed consent for publication of their clinical details and/or clinical images was obtained from the patients (CAAE: 92689218.8.0000.5404).

Subjects were evaluated according to four groups: *1*) active TED group (*n* = 17, CAS ≥ 3/7, 5 males and 12 females, aged 53 ± 14.34); *2*) inactive TED group (*n* = 16, CAS < 3, 3 males and 13 females, aged 50.13 ± 15.44); *3*) GD without apparent TED group (*n* = 16, 5 males and 11 females, aged 48 ± 14.52); *4*) healthy controls group with normal thyroid function and from an iodine sufficient area (*n* = 16, 4 males and 12 females, aged 49.63 ± 12.48). Subjects with active and inactive TED included in the study presented the onset of the eye disease simultaneously with the thyroid disease; therefore, the duration of both was similar. In addition, most of these subjects were classified as mild, and only two patients had moderate-to-severe TED who underwent examination and blood sample collection before starting glucocorticosteroid therapy. Exclusion criteria were any acute infectious or inflammatory disease, patients on hypothyroidism, patients with malignant tumors, glucocorticosteroids use, previous orbital radiotherapy, and other acute or chronic ocular surface diseases.

### Clinical Assessment

The diagnosis of Graves’ disease was established by an endocrinologist based on typical symptoms of hyperthyroidism and biochemical data. Serum thyroid-stimulating hormone (TSH), free thyroxine (fT4), free triiodothyronine (fT3), and TSH receptor antibodies (TRAbs) were determined by electrochemiluminescence immunoassay methods. The reference range value of TSH is 0.30–4.2 uUI/mL, fT4 is 0.9–1.7 ng/dL, fT3 is 0.2–0.44 ng/dL, and TRAb is < 1.58 UI/L.

Clinical eye evaluation was elaborated to define the presence of TED and the degree of inflammation based on the clinical activity score recommended by the European Group on Graves’ Orbitopathy (1). Proptosis was assessed with an ophthalmometer routinely used in our service, which measures the distance between the outer corner of the eye and the cornea.

### Ophthalmological Assessment

Patients underwent careful ophthalmological anamnesis and examination, which included the Ocular Surface Disease Index (OSDI) questionnaire (12), tear MMP-9 levels measured by the InflammaDry strip test (Rapid Pathogen Screening, Inc, Sarasota, FL) (13), and ocular surface assessment by objective measurements and phtodocumentation using Keratograph 5 M (OCULUS Optikgerate GmbH, Wetzlar, Germany) along with a comprehensive ophthalmological evaluation. The ocular surface parameters analyzed were meibography, tear meniscus height, noninvasive tear film breakup time (NITBUT), conjunctival hyperemia graded from 0 to 3 (0—absent; 1—mild; 2—moderate; and 3—severe), Schirmer test without anesthesia, sodic fluorescein, and lissamine (14). All procedures were performed by the same ophthalmologist following a preestablished protocol.

Patients were diagnosed with OSD and subclassified as aqueous tear deficiency (ATD), meibomian gland dysfunction (MGD), or mixed dry eye (MDE) based on the Tear Film and Ocular Surface Society guidelines, Dry Eye Workshop II and the International Workshop on Meibomian Gland Dysfunction (14).

### Cytokines

#### Sample preparation.

Peripheral blood samples were obtained on the morning of the assessment between 9:00 AM and 12:00 PM. The samples were centrifuged at 3,000 rpm for 10 min right after the collection, and the plasma was frozen and stored at −80°C until cytokine measurements were performed.

#### Measurement of serum IL-17A, MMP-2, MMP-3, and MMP-9.

Cytokine levels were measured in a blinded manner (e.g., clinical data were not known when the measurements were performed) by a multiplex bead assay (Thermo Fisher Scientific Human Procartaplex Mix & Match) according to the manufacturer’s instructions, on Bio-plex 200 equipment. Data were analyzed with the Bio-plex Manager 6.0 software. Values were expressed as pg/mL for all cytokines. Samples were diluted eightfold just on MMP-2, MMP-3, and MMP-9.

### Statistical Analysis

Statistical analyses were conducted using the statistical analysis system (SAS) (System for Windows, v. 9.4. SAS Institute Inc., 2002–2012, Cary, NC). Data are shown as means ± SD, median, and minimum and maximum values. Descriptive analysis with frequency tables was performed for categorical variables as well as position and dispersion measurements for continuous variables. The Chi-square or Fisher’s exact tests were used to compare proportions. The Mann–Whitney and Kruskal–Wallis tests were applied to compare continuous measures between 2 and 3 groups, respectively, followed by the Dunn test to locate differences. To assess the relationship between numerical variables, Spearman’s linear correlation coefficient was used. The significance level was set at *P* < 0.05. The power in the comparison of groups was calculated for the MMP-9 variable (0.5467), effect size for the Kruskal–Wallis test = eta^2^ (θ^2^) = 0.13 (moderate effect).

## RESULTS

### Demographic and Clinical Variables of the Cohort

Seventeen patients with active TED, 16 with inactive TED, 16 with GD without TED, and 16 healthy controls were enrolled in this study. There were no differences regarding sex, age at diagnosis, age at evaluation, smoking habits, thyroid disease duration, follow-up, TSH, fT4, TRAb, types of treatment, and comorbidities such as hypertension, diabetes, dyslipidemia, and other autoimmune diseases among the subjects analyzed. Free triiodothyronine was lower in healthy control than in patients with GD without TED (*P* = 0.0450), as shown in Table 1.

**Table 1.** Basic characteristics, laboratory data, and treatment for hyperthyroidism among patients with Graves’ disease and healthy controls

	Active TED (CAS ≥ 3)	Inactive TED (CAS < 3)	Without TED	Healthy Controls	*P* Value
	(*n* = 17)	(*n* = 16)	(*n* = 16)	(*n* = 16)	
Baseline characteristics
Female (*n*, %)	12 (70.6%)	13 (81.3%)	11 (68.8%)	12 (75%)	0.9247
Age at evaluation, yr	53 ± 14.34	50.13 ± 15.44	48 ± 14.52	49.63 ± 12.48	0.7940
	55 (29–73)	47 (19–73)	47.50 (18–66)	50.50 (30–73)	
Age at diagnosis, yr	43.88 ± 16.79	41.88 ± 16.25	42.06 ± 15.39		0.9190
	41 (17–71)	41.50 (17–65)	43.50 (15–65)		
Smoking habits					
Nonsmoker	14 (82.4%)	14 (87.5%)	13 (81.3%)	15 (93.8%)	0.8603
Currently smoking	3 (17.6%)	2 (12.5%)	3 (18.8%)	1 (6.3%)	
Thyroid disease duration, mo	109.41 ± 118	99.31 ± 89.92	70.44 ± 83.23		0.5455
	48 (12–360)	78.50 (12–324)	42 (12–348)		
Follow-up, mo	59.35 ± 83.18	75.81 ± 83.93	44.81 ± 71.26		0.2862
	24 (12–276)	36 (12–300)	24 (12–300)		
Comorbidities					
Hypertension	8 (47.1%)	6 (37.5%)	5 (31.3%)	3 (18.8%)	0.3768
Dyslipidemia	5 (29.4%)	3 (18.8%)	2 (12.5%)		0.1224
Diabetes	3 (17.6%)	2 (12.5%)	2 (12.5%)	1 (6.3%)	0.9534
Other autoimmune diseases*		1 (6.3%)	2 (12.5%)	0 (0%)	0.3178
Laboratory characteristics
TSH at the evaluation, uUI/mL	1.60 ± 1.79	1.38 ± 1.62	1.77 ± 1.44	2.55 ± 1.24	0.0846
	1.09 (0.01–4.64)	0.74 (0.01–4.77)	1.46 (0.01–4.42)	1.98 (1.11–4.87)	
fT4 at the evaluation, ng/dL	1.69 ± 1.45	1.80 ± 1.29	1.50 ± 0.76	1.11 ± 0.13	0.1166
	1.14 (0.74–7)	1.36 (0.77–5.04)	1.31 (0.77–3.79)	1.11 (0.91–1.40)	
fT3 at the evaluation, ng/dL	0.58 ± 0.87	0.51 ± 0.49	0.48 ± 0.37	0.28 ± 0.04	0.0450^a^
	0.32 (0.22–3.88)	0.32 (0.23–1.94)	0.32 (0.26–1.36)	0.27 (0.22–0.37)	
TRAb (>1.58 UI/L)	19.80 ± 16.09	25.07 ± 14.05	16.08 ± 13.87		0.2948
	11.9 (1.9–40)	26.54 (2.81–40)	9.15 (2.05–40)		
Thyroid treatment
Radioiodine	3 (17.6%)	5 (31.3%)	3 (18.8%)		0.6896
Antithyroid drug	12 (70.6%)	9 (56.3%)	11 (68.8%)		0.6465
Thyroidectomy	1 (5.9%)	2 (12.5%)	1 (6.3%)		0.8357
Levothyroxine replacement	2 (11.8%)	7 (43.8%)	4 (25%)		0.1157

Values are reported as means, median, or counts. The *P* value <0.05 denotes that statistically significant differences were found between groups. CAS, clinical activity score; fT3, free triiodothyronine; fT4, free thyroxine; *n*, number; TED, thyroid eye disease; TRAb, TSH receptor antibody; TSH, thyroid-stimulating hormone.

*Vitiligo, celiac disease, and rheumatoid arthritis. ^a^Healthy Controls < Active TED, Inactive TED and Without TED.

The active TED group was reported to have a higher CAS than the inactive TED (*P* < 0.001). The active TED group presented higher proptosis than GD without TED and healthy controls (*P* < 0.0001). In comparison, the inactive TED group demonstrated a higher proptosis than GD without TED, and the last had higher proptosis than healthy controls (*P* < 0.0001). The active TED presented more intense conjunctival redness than GD without TED (*P* = 0.0214). OSDI was higher in the inactive and active TED groups than in healthy controls (*P* = 0.0037); higher rates of dry eye disease (DED) were found in inactive TED compared with healthy individuals (*P* = 0.0113), with MGD being the most common cause of DED among these patients (62.5% in the inactive TED group vs. 6.7% among healthy control; *P* = 0.0273). The tear MMP-9 test was more frequently positive in the active TED than in other groups (*P* < 0.0001), as shown in Table 2.

**Table 2.** Ophthalmological assessment among patients with Graves’ disease

	Active TED (CAS ≥ 3)	Inactive TED (CAS < 3)	Without TED	Healthy Controls	*P* Value
	(*n* = 17)	(*n* = 16)	(*n* = 16)	(*n* = 16)	
CAS	3.76 ± 1.09	0.88 ± 0.81			<0.001^a^
	3 (3–7)	1 (0–2)			
Ophthalmometry right eye, mm	15.24 ± 3.63	13.06 ± 1.24	9.25 ± 1.0	8.88 ± 1.09	<0.0001^b^
	14 (11–22)	13 (10–15)	9.5 (8–11)	8 (8–11)	
Ophthalmometry left eye, mm	14.59 ± 4.86	13.06 ± 1.39	9.25 ± 1.0	8.88 ± 1.09	<0.0001^b^
	13 (9–25)	13 (10–15)	9.5 (8–11)	8 (8–11)	
Tear MMP-9 right eye (positive)	16 (94.1%)	12 (75%)	9 (56.3%)	2 (12.5%)	<0.0001^c^
Tear MMP-9 left eye (positive)	16 (94.1%)	11 (68.8%)	9 (56.3%)	2 (12.5%)	<0.0001^c^
OSDI	43.37 ± 27.04	36.89 ± 24.59	20.44 ± 17.74	16.02 ± 18.92	0.00373^d^
	45.45 (0–86.36)	31.63 (11.36–89.58)	12.50 (0–50)	8.33 (0–61.36)	
Diagnosis of DED	11 (68.8%)	14 (87.5%)	7 (46.7%)	5 (33.3%)	0.0113^e^
Subtypes of DED					
ATD	2 (12.5%)	1 (6.3%)	2 (13.3%)	1 (6.7%)	0.0273^f^
MGD	6 (37.5%)	10 (62.5%)	2 (13.3%)	1 (6.7%)	
MDE	3 (18.8%)	3 (18.8%)	3 (20%)	3 (20%)	

Values are reported as means, median, or counts. The *P* value <0.05 denotes that statistically significant differences were found between groups. ATD, aqueous tear deficiency; CAS, clinical activity score; DED, dry eye disease; GD, Graves’ disease; MDE, mixed dry eye; MGD, meibomian gland dysfunction; MMP-9, matrix metalloproteinase 9; *n*, number; OSDI, Ocular Surface Disease Index; TED, thyroid eye disease.

^a^Mann–Whitney test. Active TED >inactive TED;

^b^Kruskal–Wallis test followed by Dunn’s test to assess differences. Active TED > GD without TED. Active TED > healthy controls. Inactive TED > GD without TED. GD without TED > healthy controls.

^c^Chi-square test. Active TED >other groups;

^d^Kruskal–Wallis test followed by Dunn’s test to assess differences. Active TED >healthy controls; inactive TED >healthy controls;

^e^Chi-square test. Inactive TED >healthy controls;

^f^Fisher’s exact test. Inactive TED >healthy controls.

### Serum Cytokine Analysis between Groups

**Figure 1.**
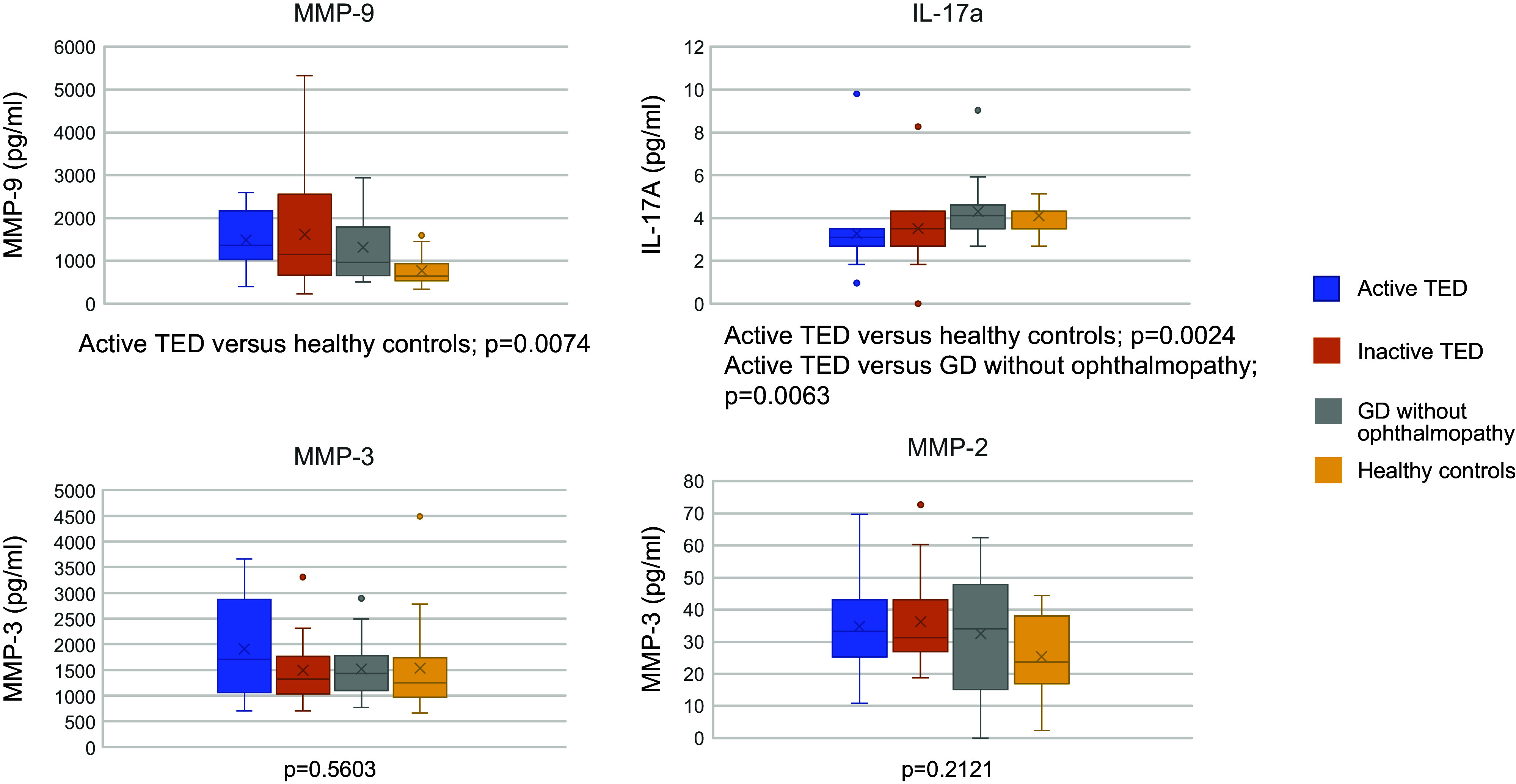
Comparison of serum cytokine levels among patients with active thyroid eye disease (TED), inactive TED, Graves’ disease (GD) without TED, and healthy controls. Serum cytokine levels are expressed in pg/mL.

There were differences in the MMP-9 and IL-17A levels among groups. The active TED group showed higher MMP-9 than healthy controls [1,919.05 ± 1,889.03 and 1,366.76 (395–8,806.80) pg/mL vs. 768.43 ± 350.66 and 640.11 (331.38–1,593.54) pg/mL; *P* = 0.0074], whereas IL-17A levels were lower among these patients as compared with patients with GD and without TED [3.26 ± 1.82 and 3.09 (0.96–9.8) pg/mL vs. 4.30 ± 1.52 and 4.12 (2.68–9.04) pg/mL; *P* = 0.0063] and healthy controls [3.26 ± 1.82 and 3.09 (0.96–9.8) pg/mL vs. 4.11 ± 0.70 and 4.32 (2.68–5.12) pg/mL; *P* = 0.0024]. No differences were found with MMP-3 and MMP-2 concentrations, as shown in Fig. 1.

### Cytokines Analysis between Groups according to Demographic, Laboratory, Thyroid Treatment, and Ophthalmological Assessment Characteristics

We found differences in MMP-9, MMP-3, and IL-17A levels regarding demographic data and thyroid disease treatment, as described in Table 3.

**Table 3.** Serum cytokines analysis between groups according to demographic data and thyroid treatment

	Active TED (CAS ≥ 3)	*P* Value	Inactive TED (CAS < 3)	*P* Value	Without TED	*P* Value	Healthy Controls	*P* Value
	(*n* = 17)		(*n* = 16)		(*n* = 16)		(*n* = 16)	
MMP-3, pg/mL
Sex
F	1,536.96 ± 782.60 1,202.28 (702.20–3,098.90)	0.0136	1,473.56 ± 674.30 1,318.80 (705.77–3,307.27)	0.6375	1,411.97 ± 521.95 1,150.44 (766.61–2,494.30)	0.1259	1,112.85 ± 262.14 1,082.72 (659.32–1,493.35)	0.0011
M	2,810.52 ± 704.51 2,868.21 (1,758.46–3,663.07)		1,573.32 ± 664.151,391.55 (1,018.98–2,309.43)		14,400.59 ± 28,153.55 1,638.42 (1,150.44–64,749.70)		2,801.14 ± 1,196.21 2,446.01 (1,821.04–4,491.47)	
Smoking
Yes	2,219.29 ± 1,267.31 1,704.05 (1,290.76–3,663.07)	0.5088	1,850.49 ± 649.04 1,850.49 (1,391.55–2,309.43)	—	23,094.36 ± 36,080.05 2,894.96 (1,638.42–64,749.70)	0.0434	2,112.33 ± —	
No	1,845.59 ± 915.25 1,652.50 (702.20–3,144.38)		1,441.09 ± 659.14 1,303.78 (705.77–3,307.27)		1,403.97 ± 484.48 1,150.44 (766.61–2,494.30)		1,496.43 ± 972.62 1,179.84 (659.32–4,491.47)	
MMP-9, pg/mL
Dyslipidemia
Yes	1,255.00 ± 655.74 1,066.26 (555.78–2,321.31)	0.1600	523.47 ± 303.58 510.96 (226.34–833.12)	0.0250	858.64 ± 315.77 858.64 (635.36–1,081.92)		768.43 ± 350.66 640.11 (331.38–1,593.54)	
No	2,208.24 ± 2,173.46 1,673.12 (395–8,806.80)		1,860.44 ± 1,442.38 1,231.59 (612.27–5,325.73)		1,378.47 ± 835.99 1,164.54 (507.11–2,938.76)			
RAI
Yes	3,829.65 ± 4,412.95 2,287.14 (395–8,806.80)	0.6765	633.62 ± 293.66 612.27 (226.34–985.42)	0.0055	812.77 ± 289.14 849.29 (507.11–1,081.92)	0.3643		
No	1,509.64 ± 610.75 1,355.72 (555.78–2,592.57)		2,053.45 ± 1,491.04 1,279.96 (621.82–5,325.73)		1,429.04 ± 844.85 1,479.79 (547.17–2,938.76)			
Methimazole
Yes	1,544.94 ± 673.46 1,471.54 (395–2,592.57)	>0.999	2,096.62 ± 1,572.72 1,279.96 (621.82–5,325.73)	0.0164	1,568.04 ± 847.47 1,494.78 (547.17–2,938.76)	0.0897		
No	2,816.93 ± 3,405.72 1,276.98 (555.78–8,806.80)		983.79 ± 898.42 774.73 (226.34–2,943.69)		753.48 ± 220.95 693.71 (507.11–1,081.92)			
Levothyroxine
Yes	1,421.46 ± 1,224.26 1,421.46 (555.78–2,287.14)		983.79 ± 898.42 774.73 (226.34–2,943.69)	0.0164	768.42 ± 252.20 742.33 (507.11–1,081.92)	0.1703		
No	1,985.40 ± 1,982.70 1,366.76 (395–8,806.80)		2,096.62 ± 1,572.72 1,279.96 (621.82–5,325.73)		1,495.18 ± 846.53 1,487.28 (547.17–2,938.76)			
IL-17A, pg/mL
Methimazole
Yes	3.61 ± 2.08 3.50 (0.96–9.80)	0.0189	3.89 ± 1.84 3.50 (1.83–8.27)	0.9131	4.56 ± 1.74 4.32 (2.68–9.04)	0.4510		
No	2.43 ± 0.38 2.68 (1.83–2.68)		3.50 ± 0.67 3.50 (2.68–4.32)		3.75 ± 0.69 3.91 (2.68–4.32)			

Values are reported as means or counts. The *P* value <0.05 denotes that statistically significant differences were found between groups. Only one individual in the control group was a smoker. F, female; IL-17A, interleukin 17 A; M, male; MMP-3, matrix metalloproteinase 3; MMP-9, matrix metalloproteinase 9; *n*, number; RAI, radioactive iodine; TED, thyroid eye disease.

In contrast, no differences were found between MMP-2, MMP-3, MMP-9, and IL-17A among each group regarding laboratory variables (TSH, fT4, fT3, and TRAb), thyroid function status at evaluation (euthyroidism vs. hyperthyroidism), ophthalmological assessment (CAS and its subitems, tear MMP-9 test, and ocular surface assessment) (data not shown).

#### MMP-9.

Serum MMP-9 levels were lower in inactive TED who underwent radioactive iodine (RAI) therapy and those on levothyroxine replacement, with a *P* value of 0.0055 and 0.0164, respectively. The opposite was shown among patients in the same group who were on antithyroid drug therapy with methimazole as they presented higher levels of MMP-9 (2,096.62 ± 1,572.72 vs. 983.79 ± 898.42; *P* = 0.0164). MMP-9 concentrations were lower among patients with inactive TED, formally diagnosed with dyslipidemia and on HMG-CoA (3-hydroxy-3-methyl glutaryl-CoA) reductase inhibitors (523.47 ± 303.58 vs. 1,860.44 ± 1,442.38; *P* = 0.0250).

#### MMP-3.

Serum MMP-3 levels were higher in men than women in the active TED group (2,810.52 ± 704.51 vs. 1,536.96 ± 782.60; *P* = 0.0136) and healthy controls (2,801.14 ± 1,196.21 vs. 1,112.85 ± 262.14; *P* = 0.0011). MMP-3 was also higher among current smokers in the GD without TED group (*P* = 0.0434), which was not reported in other groups.

#### IL-17A.

Serum IL-17A levels were higher in the active TED group who were on antithyroid drug therapy with methimazole (3.61 ± 2.08 vs. 2.43 ± 0.38, with a *P* value of 0.0189).

### Spearman’s Correlation

#### Serum cytokines and demographic data.

A negative correlation was found between age at assessment and MMP-9 levels among patients with inactive TED (ρ = −0.6343; *P* = 0.0083). Regarding the follow-up time, we found a robust negative correlation with MMP-2 levels among GD without TED (ρ = −0.64; *P* = 0.0102) and MMP-9 levels among inactive TED (ρ = −0.61; *P* = 0.0108) (Table 4).

**Table 4.** Spearman’s correlation between MMP-2, MMP-3, and MMP-9 and demographic, laboratory, and ophthalmological parameters

		Active TED		Inactive TED		GD without TED		Healthy Controls	
Demographic		ρ	*P* Value	ρ	*P* Value	ρ	*P* Value	ρ	*P* Value
Age at evaluation	MMP-9	−0.3008	0.2407	−0.6343	0.0083	−0.3336	0.2067	−0.2708	0.3104
Follow-up, mo	MMP-2	0.0759	0.7798	−0.0705	0.8028	−0.64	0.0102		
	MMP-9	0.4587	0.0640	−0.6173	0.0108	−0.1934	0.4729		

Laboratory		*ρ*	*P* Value	*ρ*	*P* Value	*ρ*	*P* Value	*ρ*	*P* Value
fT3	MMP-9	−0.1338	0.6086	0.3387	0.1994	0.6834	0.0035	−0.208	0.9390
TRAb	MMP-9	−0.2468	0.3397	−0.5314	0.0341	−0.5605	0.0239		

Ophthalmological parameters		ρ	*P* Value	ρ	*P* Value	ρ	*P* Value	ρ	*P* value
Ophthalmometry	MMP-2	−0.1007	0.7105	−0.1278	0.6498	−0.5374	0.0388	0.3027	0.2545
	MMP-9	−0.1668	0.5223	0.5158	0.0408	−0.1415	0.6012	0.0957	0.7244
Redness	MMP-3	0.6736	0.0042	0.1740	0.5192	0.1665	0.5531	0.5565	0.0312
Schirmer test	MMP-2	−0.2234	0.4235	0.2604	0.3487	−0.6302	0.021	−0.2590	0.3513

The *P* value <0.05 denotes that statistically significant correlations were found. fT3, free triiodothyronine; GD, Graves' disease; MMP-2, matrix metalloproteinase 2; MMP-3, matrix metalloproteinase 3; MMP-9, matrix metalloproteinase 9; TED, thyroid eye disease; TRAb, TSH receptor antibody.

#### Serum cytokines and laboratory data.

fT3 was positively correlated with MMP-9 levels in the GD without TED group (ρ = 0.6834, *P* = 0.0035). Interestingly, TRAb was negatively correlated with MMP-9 levels in subjects from inactive and GD without TED groups (ρ = −5314, *P* = 0.0341 and ρ = −0.5605, *P* = 0.0239). No correlations between other cytokines concentrations and TSH, fT4, fT3, and TRAb were reported in other groups (Table 4).

#### Serum cytokines and ophthalmological assessment.

Values obtained with ophthalmometry were negatively correlated with MMP-2 among GD without TED (ρ = −0.5374, *P* = 0.0388). On the other hand, we found a positive correlation between ophthalmometry values and MMP-9 in the inactive TED group (ρ = 0.5158, *P* = 0.0408). Redness was positively correlated with MMP-3 levels among subjects with active TED (ρ = 0.6736, *P* = 0.0042) and healthy controls (ρ = 0.55, *P* = 0.0312). There was a negative correlation between the Schirmer test and MMP-2 levels in the GD without TED group (*r* = −0.6302, *P* = 0.021) (Table 4).

## DISCUSSION

This study demonstrated that MMP-9 serum levels were increased among patients with active TED compared with healthy controls, showing a possible link between this MMP and acute orbital inflammation. In addition, it was related to radioactive iodine treatment, longer follow-up time, and higher values in ophthalmometry among patients with inactive TED as well as thyroid function among GD without TED. It is important to emphasize that it would be expected that MMP-9 to be different between the groups with and without TED, possibly distinguishing the ones with active and inactive TED; however, MMP-9 was elevated in all three groups with GD. These findings suggest that MMP-9 may be involved in the active phase of ophthalmopathy and the active phase of inflammation related to GD.

MMPs are involved in degradation and ECM remodeling through a delicate balance with their counteracting tissue inhibitors of metalloproteinases (7, 8). Their importance has already been described in several conditions, including cancer, wound healing process, cardiovascular disease, and other autoimmune disorders such as rheumatoid arthritis (8). In the setting of active TED, we reported that serum levels of MMP-9 were higher among these patients than in healthy controls, which agrees with previous studies. Moreover, they also presented more frequent positive tear MMP-9 tests than all other groups. The MMP-9 synthesis is controlled by several factors, including IL-1β, which is predominantly released in the Th1 immune response, explaining their possible relationship in the acute inflammatory phase of TED, which differs from the TRAb influence in the Th2 immune response (8, 10).

Besides its role in active TED, we found that serum MMP-9 was reduced in patients in the inactive TED group who underwent RAI and were on levothyroxine replacement. In contrast, it was elevated among the patients on antithyroid drugs, such as methimazole. MMP-9 was also negatively correlated with follow-up time and age at assessment among inactive TED and positively correlated with fT3 in GD without TED. We hypothesize that patients on antithyroid drugs, with higher fT3 levels and shorter follow-up time, are probably in the active phase of GD and, therefore, under more inflammation. In contrast, the ones who were on levothyroxine replacement underwent RAI, and older patients (higher age at evaluation) were in a chronic phase, explaining the correlations found. Unexpectedly, the study showed a negative correlation between TRAb and MMP-9 concentrations in inactive TED and GD without TED groups, which does not agree with the findings from Kapelko-Stowik et al. (10). On the other hand, some authors have reported a similar negative correlation, yet between TRAb and RANTES (15) and IL-7 and fT4 (16). The mechanisms behind the negative correlation between TRAb and MMP-9 remain unclear.

As to OSD, MMP-9 concentrations were not related to the diagnosis of DED but were positively correlated with proptosis in patients with inactive TED in a chronic and fibrotic phase. Another interesting finding that possibly contributes to our hypothesis that MMP-9 is linked to systemic inflammation was lower MMP-9 levels among patients with inactive TED formally diagnosed with dyslipidemia and on HMG-CoA reductase inhibitors. It has been suggested that hypercholesterolemia may be an additional risk factor for TED (17, 18), and statins may protect against the development of TED in newly diagnosed patients with GD (19). Adding atorvastatin to the classic treatment for active TED may also improve outcomes (20).

With regard to MMP-3, it was found to be positively correlated with redness in subjects with active TED, which agrees with our hypothesis that MMPs, especially MMP-9, may be involved in the acute inflammatory phase of TED. We also reported that the mean MMP-3 concentrations were higher among smokers in all groups; however, it was associated with the group of current smokers with GD without TED. It is well known that smoking is a crucial risk factor associated with the more severe thyroid disease, as well as the development and deterioration of TED, mainly activating inflammation, adipogenesis, and fibrosis. It has been previously reported that tissue hypoxia may increase IL-1β, probably modifying MMP synthesis (21, 22). We hypothesized that MMP-3 might be more related to thyroid disease than ophthalmopathy.

Another finding that has not been described in previous studies is that MMP-3 was higher among male than female subjects in the active TED and healthy control groups. We could speculate that male subjects with active TED tend to present a more severe ophthalmopathy and, consequently, more intense inflammation and elevated MMP-3 levels (23). It could also be explained by the finding that estrogens and progesterone are among several factors able to suppress excess MMP secretion (24). For that reason, women would present lower MMP levels.

Regarding MMP-2, we found interesting correlations in the group with GD without TED. A shorter follow-up period was shown to be negatively correlated with MMP-2 concentrations, which may reflect newly diagnosed patients in the active inflammatory phase of the disease. With respect to the parts of the ophthalmological assessment, ophthalmometry and the Schirmer test were also negatively correlated with MMP-2 concentrations. We speculate that MMP-2, similar to MMP-9 and MMP-3, is involved in acute inflammation; therefore, patients with a better Schirmer test profile (better tear production) are expected to be less inflamed. The mechanism behind the correlation with ophthalmometry remains unclear.

IL-17A, a proinflammatory cytokine produced by Th17 cells, has been involved in GD with and without TED. Most studies have evidenced higher IL-17 concentrations in patients with TED, positively correlating with the disease activity (5, 6). Kim et al. (5) reported higher levels of IL-17 in patients with TED than in healthy controls and between active TED and inactive TED; however, this study did not include data on GD without TED. Wei et al. (6) assessed the levels of IL-17 in patients with active and inactive TED, GD without TED, and healthy control. They reported that all patients with GD with and without TED presented higher levels of IL-17 than healthy control, and similar to the previous study (5), IL-17 was also higher among active TED than inactive TED and GD without TED.

Our results differed from those of the previous studies, as we described higher concentrations of IL-17A among GD without TED and healthy controls than in the active TED group, which is somewhat similar to the findings described by Shen et al. (25). They reported that patients with active TED, inactive TED, and GD without TED presented higher IL-17 levels than healthy control, indicating that IL-17 might be related to the fibrotic process seen in the chronic phase of TED. However, both active and inactive TED groups had lower levels of IL-17 than GD without TED, suggesting that IL-17 tends to reduce as the eye disease develops. Given that the role of IL-17A is yet to be defined, more evidence is needed to establish the link between IL-17A and ophthalmopathy. In addition to the previous finding, we described that IL-17A levels were elevated among all patients on methimazole; however, it was only significant in the active TED group, possibly reflecting a relationship with the active inflammatory phase of both hyperthyroidism and TED or perhaps a stimulatory effect of methimazole itself on IL-17.

Some limitations in our study must be pointed out. First, the small sample sizes might explain some of the unexpected findings. Second, the evaluation of serum cytokines using multiplex immunoassays is known to be less robust due to potential interactions with multiple different antibodies and proteins. Finally, the cross-sectional character of the present study. On the other hand, our study thoroughly compared patients in two distinct phases of the inflammatory process, active and nonactive TED, along with a group without apparent TED and healthy control.

### Conclusions

In conclusion, MMP-9 was elevated in GD regardless of the presence or absence of TED, and the phase of ocular inflammatory activity suggests that MMP-9 may be involved in the active inflammatory phase of ophthalmopathy as well as in the active phase of GD. The central question is whether these MMPs are a potential target for future treatments since fewer studies have reported the clear role of MMPs in the active and chronic phases of inflammation in GD. Further studies are needed to establish the precise role of MMPs in GD with and without TED.

## DATA AVAILABILITY

Data will be made available upon reasonable request.

## GRANTS

DEZ-W had a National Council of Technological and Scientific Development Scholarship (CNPq) (303068/2021-3) and FAEPEX (Fundo de Auxílio a Pesquisa da Unicamp) No. 92867-20.

## DISCLOSURES

No conflicts of interest, financial or otherwise, are declared by the authors.

## AUTHOR CONTRIBUTIONS

C.M.R., E.B.B., F.R., M.A., and D.E.Z.-W. conceived and designed research; C.M.R., E.B.B., and F.R. performed experiments; C.M.R., E.B.B., M.A., and D.E.Z.-W. analyzed data; C.M.R., E.B.B., F.R., M.A., and D.E.Z.-W. interpreted results of experiments; C.M.R. and E.B.B. prepared figures; C.M.R. and E.B.B. drafted manuscript; C.M.R., E.B.B., C.C.A., F.R., M.A., and D.E.Z.-W. edited and revised manuscript; C.M.R., E.B.B., F.R., M.A., and D.E.Z.-W. approved final version of manuscript.
